# Hemiplegia

**Published:** 1846-02

**Authors:** 


*Hemiplegia.*—Dr. Foster reported a case of hemiplegia in a child during dentition: he had lost the use of the left leg entirely, but had the partial use of the left arm. The treatment consisted in the use of stimulants, applied externally; under which the limb had been restored.—*Ibia*

